# Content and Face Validity of the Mackey Childbirth Satisfaction Rating Scale Questionnaire Cross-culturally Adapted to Brazilian Portuguese

**DOI:** 10.1055/s-0039-1692125

**Published:** 2019-06

**Authors:** Fernanda Lopes, Nelson Carvas Júnior, Mary Uchiyama Nakamura, Roseli Mieko Yamamoto Nomura

**Affiliations:** 1 Department of Obstetrics, Escola Paulista de Medicina, Universidade Federal de São Paulo, São Paulo, SP, Brazil; 2 Postgraduate program in Health Sciences, Instituto de Assistência Médica ao Servidor Público Estadual, São Paulo, SP, Brazil

**Keywords:** childbirth, patient satisfaction, questionnaires, validation studies, parto, satisfação do paciente, questionários, estudos de validação

## Abstract

**Objective** The aim of this study was to determine the content and face validity of the Mackey Childbirth Satisfaction Rating Scale (MCSRS) questionnaire cross-culturally adapted to Brazilian Portuguese.

**Methods** The MCSRS is a questionnaire with 34 items related to childbirth satisfaction. The forward- and back-translated versions were compared with the original material, and 10 experts analyzed each item according to the following criteria: clarity, semantic equivalence, appropriateness, and cultural relevance. The final version was presented to 10 mothers for face validation to ensure the questionnaire would suit the target population.

**Results** The total of 34 items assessed by experts for clarity, semantic equivalence, appropriateness, and relevance showed positive agreement of 0.85, 0.92, 0.97 and 0.97; negative agreement of 0.13, 0.09, 0.04 and 0.04; and total agreement of 0.75; 0.85, 0.94 and 0.94, respectively. Multilevel linear modeling was applied with crossed random effects and with nested random effects for each judge. The intercept of each criterion was as follows: clarity, 0.87; semantic equivalence, 0.92; appropriateness, 0.96; and cultural relevance, 0.96. The overall mean of agreement was 92.8%. The face validity measurement yielded 80% of agreement on the items, all of them clearly understood.

**Conclusion** The final version of the Brazilian Portuguese MCSRS questionnaire had face and content validity confirmed. This instrument of evaluation of maternal satisfaction during childbirth was validated to be applied in the Brazilian female population.

## Abstract (pt)

**Resumo:**

**Objetivo** O objetivo deste estudo foi determinar a validação de face e conteúdo da Escala Mackey de Avaliação da Satisfação com o Parto (MCSRS, na sigla em inglês), transculturalmente adaptada para o português brasileiro.

**Métodos** O MCSRS é um questionário com 34 itens relacionados à satisfação com o parto. As versões traduzida e retrotraduzida foram comparadas com o material original, e 10 especialistas analisaram cada item de acordo com os seguintes critérios: clareza, equivalência semântica, pertinência e relevância cultural. A versão final foi apresentada a 10 mães para validação de face, para garantir que o questionário fosse adequado à população-alvo.

**Resultados** O total de 34 itens avaliados por especialistas para clareza, equivalência semântica, pertinência e relevância apresentou concordância positiva de 0,85, 0,92, 0,97 e 0,97; concordância negativa de 0,13, 0,09, 0,04 e 0,04; e concordância total de 0,75; 0,85, 0,94 e 0,94, respectivamente. A modelagem linear multinível foi aplicada com efeitos aleatórios cruzados e com efeitos aleatórios aninhados para cada juiz. A intercepção de cada critério foi a seguinte: clareza, 0,87; equivalência semântica, 0,92; pertinência, 0,96; e relevância cultural, 0,96. A média geral de concordância foi de 92,8%. A mensuração da validade de face foi 80% de concordância nos itens, todos claramente entendidos.

**Conclusão** A versão final do questionário MCSRS do português brasileiro teve validade de face e conteúdo confirmada. Este instrumento de avaliação da satisfação materna durante o parto foi validado para ser aplicado na população feminina brasileira.

## Introduction

Maternal satisfaction during childbirth is not always achieved, and many factors may influence this experience. Therefore, understanding women's satisfaction with their experience of childbirth is relevant to community health care providers and can be an indicator of the quality of maternity care.

Patient satisfaction is a multidimensional concept that has received widespread research attention. It has been evaluated from different perspectives and with different objectives^1^ but without a standard for the psychometric properties.^2^ Patient satisfaction has been characterized as a reflection of preference, expectations, and the reality of care.^3^ Women's satisfaction with childbirth should be investigated using questionnaires developed for this specific purpose, cross-culturally adapted and validated for the results to be globally comparable when the same tool is applied. Cultural adaptation aims at accomplishing content equivalence to ensure the utmost similarity at the conceptual level in both cultures, that is, the culture of the original analytical tool and that of the new version. Further adjustments are made for adequate fulfillment of the needs of the new population, language or place, or any combination thereof. Language can also be altered to better reflect current social realities.^4^

Most women experience childbirth without any complications, but maternal satisfaction at such a time is not always felt. Recent studies were conducted to validate questionnaires in different languages investigating women's satisfaction with childbirth.^3^ ^5^ ^6^ However, Brazilian Portuguese was not included. The Mackey Childbirth Satisfaction Rating Scale (MCSRS)^7^ was developed in the United States and is a complete scale encompassing the most important factors related to women's satisfaction. This questionnaire contains 34 self-reported items divided into six subscales: self, baby, nurse, partner, physician, and an overall labor and delivery evaluation.

The MCSRS showed that satisfaction depends on complex factors, namely a positive relationship with the medical team, control over the situation, and, most importantly, fulfillment of their expectations. Personal control during childbirth was an important factor related to the women's satisfaction with the childbirth experience.^7^ Percentages of both ‘satisfied’ and ‘very satisfied’ on the MCSRS in Belgium and the Netherlands, in general, was 47.3%.^8^ The satisfaction and perceived control in childbirth was studied using MCSRS in a large sample of women giving birth at three public hospitals in Egypt, Lebanon and Syria. The findings revealed a high level of satisfaction and an average level of perceived control in labor in each country and in the overall sample of women giving birth at the three public hospitals.^9^ The validation of this scale in different cultures demonstrates the universality of the application of this method, which can be used both in research and in health impact assessments.

The purpose of this study was to perform the cross-cultural adaptation of the MCSRS to Brazilian Portuguese and determine the face validity of the version for Brazilian community.

## Methods

### The Questionnaire

The MCSRS is a 34-item questionnaire related to childbirth satisfaction. It comprises the following 5 sub-scales: self (9 items; Q3–Q11); partner (2 items; Q12 and Q13); baby (3 items; Q14–Q16); nurse (8 items; Q: 17, 19, 21, 23, 25, 27, 29, 31, 33); and physician (8 items; Q: 18, 20, 22, 24, 26, 28, 30, 32). Additionally, it contains a subscale for global overall labor and delivery evaluation (2 items). The degree of satisfaction or dissatisfaction with each item was indicated on a 5-point Likert scale as follows: “very dissatisfied,” unsatisfied,” “neither satisfied nor unsatisfied,” “satisfied,” and “very satisfied.” Question 35 is an open-ended question about the factors that contributed to satisfaction and dissatisfaction. Question 36 asks the respondent to list the factors of the previous question in order of importance. The last four items are about the general perception of the respondent with respect to birth expectations and to the experience as primarily positive or negative.

### Translation, Back-translation and Cultural Adaptation

To develop the Brazilian Portuguese version of the MCSRS, formal permission was obtained from Marlene C. Mackey by e-mail, as is commonly performed in consideration of the authorship of the instrument. A forward–backward procedure was applied. The original questionnaire was first converted into Brazilian Portuguese by a bilingual physician who was living in the United States and was specialized in obstetrics and gynecology. The translator was asked to aim for a conceptual rather than a literal translation. Afterwards, a native English speaker and professional translator, who was completely blinded to the original questionnaire, converted the Portuguese translation back into the English language. Next, the original English text and the back-translation were compared to detect inconsistencies, mistranslations, changes in meaning, cultural gaps and/or lost words or phrases.^10^ The back-translation was not sent to the original author of the MCSRS because the cultural differences are considered relevant.

### Content Validity by Experts

Content was validated by the following 10 experts: 1 obstetrician, 4 obstetricians who were also professors of obstetrics, 4 midwives (nurses) who were also professors of midwifery, and a psychologist, who was also a professor of his specialty. All of these judges were experienced in providing health care for pregnant women. Initially, they analyzed the first translation into Portuguese. The structures of the statements were examined and discussed, and each item was evaluated according to the following aspects: clarity, semantic equivalence, appropriateness, and cultural relevance. Next, the original questionnaire, its first translation into Portuguese, and the back-translation were compared before the final version of the questionnaire in Portuguese could be written. In general, the structure and content of the questionnaire originally translated into Portuguese remained unchanged, except for one modification to explain the term ‘delivery,’ which received an explanation in brackets (“The moment the baby was expelled”) to adapt it to the Brazilian culture.

### Face Validity

As a strategy to evaluate face validity and to ensure the questionnaire would suit the target population, a draft of the final version of the Brazilian Portuguese questionnaire was presented to 10 mothers. Face validity was performed with the aim to verify the ability of this instrument to be understandable and relevant for the targeted population, and generally includes a pilot testing.^11^ We invited low-risk postpartum women with a single pregnancy and live birth, in their second day after delivery and not yet discharged, aged between 18 and 34 years, and whose educational level allowed them to understand the questionnaire. They were oriented to read the items and assess them for clarity of wording and understanding. Face validity is a measure of usability; thus, an evaluation question was included to determine the ease of comprehension of each item on an ordinal scale with alternatives according to the 5-point Likert scale: (1) I did not understand anything, (2) I understood very little, (3) I understood it reasonably well; (4) I understood it well; (5) I understood it very well and I have no questions. This procedure constitutes an aspect of face validity, that is, the ability of the subjects who evaluate the instrument to understand the items as stated in the questionnaire.

### Statistical Analysis

In content analysis, judges should be experts in the construct, and according to Pasquali, a minimum of six judges will be sufficient to accomplish this task.^12^ For content validity, the following tests were applied: analyses of agreement and reliability of the experts' data using gross indices of agreement; Fleiss' k coefficient of agreement between judges; and multilevel analysis of the responses, simultaneously considering variation by evaluator, item, and criterion. For the analysis of concordance among judges, the database was separated into four distinct banks, one for each of the criteria (clarity, semantic equivalence, appropriateness, and cultural relevance). To simplify the analysis, every statistic was calculated on the assumption that the complete instrument was relatively homogeneous. For the analysis of frequency of agreement, basic statistics were computed based on relative frequency, using the method described by Uebersax^13^ for reference. The agreement ratio was considered good when above 0.7 and very good when above 0.8. For the second analysis of the experts' data, we used a k index suitable for multiple simultaneous evaluators, that is, Fleiss' k, as implemented in the inter-rater reliability (IRR) package version 0.84 (R Foundation, Vienna, Austria) within the R programming language (version 3.1). Finally, the experts' assessments were analyzed simultaneously using a multilevel linear model^14^ with crossed random effects by evaluator, and nested random effects by item among the evaluation criteria. Nesting is justified because it makes little sense to assess variation between items in general insofar as the criteria are different from one another. The model can be represented by the following equations:

yi = μ + γj[i] + νk[l[i]] + i

γj ∼ Normal (0, σ2) νk

∼ Normal (ζl[k], σ2) ζl

∼ Normal (0, σ2)

i ∼ Normal (0, σ2)

Where yi represents the response of an expert j to an item k, nested in criterium 1. The equation was presented to clarify the method. Although the normal model is not the most adequate for binary data, its application facilitates the interpretation of the parameters and the calculation of the intraclass correlation coefficient (ICC) of the model. The multilevel model was adjusted with the routines of the lme4 package, version 1.1 12^15^, using the restricted maximum likelihood (REML) criterium for parameter estimation.

For face validity analysis, each of the 10 women was considered a judge with respect to the comprehensibility of the statements. Therefore, face validity was translated into analysis of agreement among participants. The questionnaire was piloted on a smaller sample of respondents, and the sample size of 10 women was sufficient to perform systematic appraisal of its performance, as stated by Rattray and Jones.^16^ We performed 2 analyses: (1) numerical analysis of the answers to the items-exploratory analysis; (2) analysis of the raw agreement indices. For the exploratory analysis, we filtered the answers to the question about the comprehensibility of the items. Then, the responses to the statements on comprehensibility were presented through the median and first and third quartiles. The agreement among the participants was evaluated by means of the proportion of specific and absolute agreement, using the method described by Uebersax.

### Ethical Considerations

This study was approved by the local Human Research Ethics Committee (CAAE: 51125515.7.0000.5505). All the participants (women and judges) were informed about the objectives of the study and assured about its confidentiality. The women participants were informed they could withdraw from the study at any time on simple request. Finally, a written informed consent was obtained from every participant.

## Results

### Translation, Back Translation and Cultural Adaptation

The forward-translated and the back-translated versions of the questionnaire are presented as supplementary data. The comparison of the two English versions (the original source material and the back translation) revealed that the versions were similar and only minor changes were necessary (supplementary data). The final version in Brazilian Portuguese was evaluated by ten experts for content validity.

### Content Validity

A total number of 34 items was assessed by the experts. The basic agreement statistics based on relative frequency for the criteria of clarity, semantic equivalence, appropriateness, and cultural relevance are presented in Table 1. The positive agreement of the judges was considerably greater than their negative agreement, which means that, on most items, judges agreed the evaluation criteria were being met. Negative agreement indicated lack of consensus among the evaluators and it was considerably low. Of all the criteria, clarity had the lowest agreement ratio (75%), although such a proportion by itself is quite high.

**Table 1:** Reliability analysis of the items in the Brazilian Portuguese version of the Mackey Childbirth Satisfaction Rating Scale questionnaire evaluated by 10 judges for agreement statistics based on relative frequency (Uebersax) and multilevel linear modeling with nested random effects for each judge

Evaluation criteria	Total agreement	Positive agreement	Negative agreement	Multilevel linear model intercept
Clarity	0.75	0.85	0.13	0.87
Equivalence	0.85	0.92	0.09	0.92
Appropriateness	0.94	0.97	0.09	0.96
Cultural relevance	0.94	0.97	0.04	0.96

For the second analysis of the experts' data, a k index for multiple simultaneous evaluators was calculated. However, it turned out it was inadequate, for it was very close to zero (semantic equivalence = 0.01; appropriateness = 0.01; cultural relevance = 0.01), becoming negative for clarity (-0.02). These results seem to contradict the raw data evaluation. The reason is the strategy used for calculating the coefficient: when there is a high level of agreement and few classification categories (as in the present case), the correction for random agreement ends up decreasing the end value. Thus, we decided not to use this coefficient. As Fleiss' k is derived from the traditional ICC, which is computed from ANOVA, we chose to analyze the variation among assessments using a multilevel model.

Multilevel linear modeling was applied with crossed random effects for each judge, and nested random effects were used. The overall agreement mean was 92.8%. Table 1 presents the model intercept for each criterium. The results are very similar to those assessing relative frequency. The criteria of appropriateness and cultural relevance elicited the most agreement, whereas the clarity criterium prompted the most discordance even if slight. With respect to the items nested in the criteria, the ICC was 0.18 and the standard deviation of the random term was 0.11. Although the ICC was not so high, the judges, in general, tended to agree on the evaluation of an item within a given criterium.

The random effects allowed identifying the items that received the most negative evaluations. For the clarity criterium, the items with the lowest random effect were Q36 (-0.33), Q33 (-0.26), and Q34 (-0.26); for the semantic equivalence criterium, the lowest items were Q14 (-0.26), Q37 (-0.22), and Q13 (-0.19); with respect to cultural relevance, the lowest average items were Q17 (-0.23), Q9 (-0,18), and Q11 (-0.11); and finally, with regard to appropriateness, the lowest items were Q17 (-0.23), Q9 (-0.18), and Q11 (-0.11).

### Face Validity

**Fig. 1:**
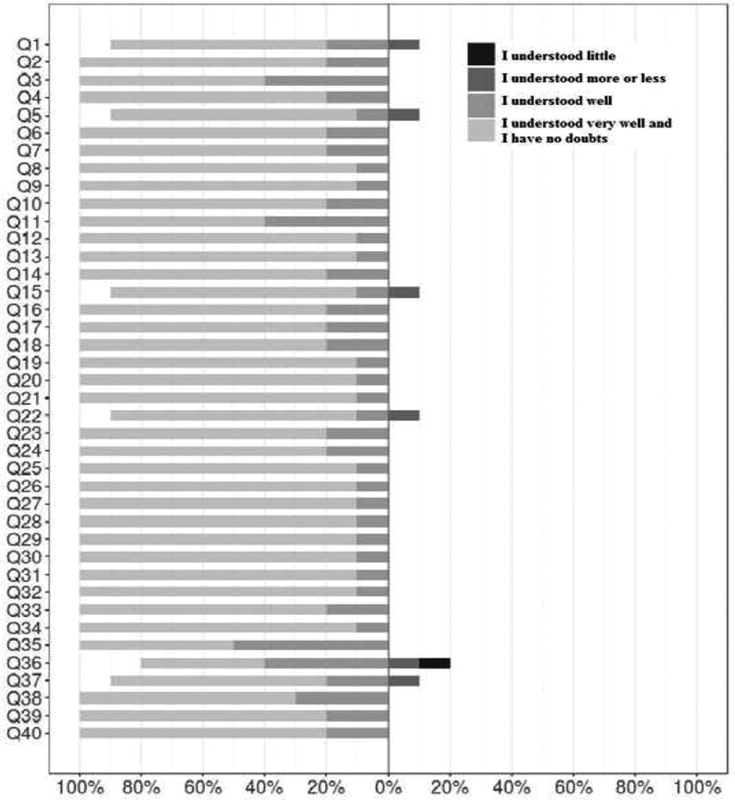
Responses to questions about item comprehensibility in the Brazilian Portuguese version of the Mackey Childbirth Satisfaction Rating Scale questionnaire.

In our study, 10 postpartum women also confirmed the face validity of the tool. Fig. 1 summarizes the data on the women's comprehension of the items in the questionnaire. There was a high proportion of answers attesting to the easy comprehensibility of the items. What is most noteworthy is that no statement was considered incomprehensible. Also relevant is the fact that “I understood very well” was the most selected answer with the two highest categories making up the totality of the responses for the 34 items. The items that elicited comparatively lower comprehension responses were the following: (Q1) “Your overall experience in labor”; (Q5) “Your ability to manage your labor contractions”; (Q15) “The amount of time that passed before you held your baby for the first time”; (Q22) “The amount of explanation or information received from the medical staff in labor and delivery”; (Q36) “Using the items you named in question 35 above, number them in order of importance. Place ‘1’ in front of the item that most contributed to your satisfaction/dissatisfaction; Place ‘2’ in front of the next most important item and so on until you number all the items”; and (Q37) “In general, was your experience in labor what you expected it to be?” The question generating the most comprehension problems was 36, even if these were isolated issues. However, since the question is about listing items, it can be rewritten to make it clearer or simpler for the respondent.

Table 2 summarizes the median score data on each item's comprehensibility. Except for items Q35 and Q36, the responses demonstrate very good comprehension (median 4.0). The first quartile's median is also 4.0 in 33 statements of the questionnaire, which again confirms the postpartum women's ability to understand the questions easily.

**Table 2:** Results of the responses to the questions related to item comprehension in the Brazilian Portuguese version of the Mackey Childbirth Satisfaction Rating Scale questionnaire (median, and first and third quartiles)

Item	1^st^ quartile	Median	3^rd^ quartile
Q1	3.2	4.0	4.0
Q2	4.0	4.0	4.0
Q3	3.0	4.0	4.0
Q4	4.0	4.0	4.0
Q5	4.0	4.0	4.0
Q6	4.0	4.0	4.0
Q7	4.0	4.0	4.0
Q8	4.0	4.0	4.0
Q9	4.0	4.0	4.0
Q10	4.0	4.0	4.0
Q11	3.0	4.0	4.0
Q12	4.0	4.0	4.0
Q13	4.0	4.0	4.0
Q14	4.0	4.0	4.0
Q15	4.0	4.0	4.0
Q16	4.0	4.0	4.0
Q17	4.0	4.0	4.0
Q18	4.0	4.0	4.0
Q19	4.0	4.0	4.0
Q20	4.0	4.0	4.0
Q21	4.0	4.0	4.0
Q22	4.0	4.0	4.0
Q23	4.0	4.0	4.0
Q24	4.0	4.0	4.0
Q25	4.0	4.0	4.0
Q26	4.0	4.0	4.0
Q27	4.0	4.0	4.0
Q28	4.0	4.0	4.0
Q29	4.0	4.0	4.0
Q30	4.0	4.0	4.0
Q31	4.0	4.0	4.0
Q32	4.0	4.0	4.0
Q33	4.0	4.0	4.0
Q34	4.0	4.0	4.0
Q35	3.0	3.5	4.0
Q36	3.0	3.0	4.0
Q37	3.2	4.0	4.0
Q38	3.2	4.0	4.0
Q39	4.0	4.0	4.0
Q40	4.0	4.0	4.0

The percentage of women who selected each answer is displayed in Table 3. The highest percentage (80.1%), as well as the most inclusive, is the 4^th^ level of understanding, that is, “I understood very well and I have no questions.” The third and next level of comprehensibility, expressed as “I understood well,” has a considerably lower selection ratio, showing there was a clear tendency among respondents toward finding some of the items somewhat less understandable than others. Finally, the categories of poor comprehensibility were never selected.

**Table 3:** Percentage of agreement of the answers related to item comprehensibility in the Brazilian Portuguese version of the Mackey Childbirth Satisfaction Rating Scale questionnaire for determining face validity

Items comprehension	Proportion of agreement (%)
I understood little	0
I understood more or less	0
I understood well	14.7
I understood very well and I have no doubt	80.1
Total	67.1

The absolute percentage of agreement is 67%, indicating that the opinions are relatively similar in terms of item comprehensibility. As participants tended to select the same statements, which are those expressing easy comprehensibility, it is possible to conclude that the face validity of this tool has been demonstrated.

## Discussion

The present study is the first to report the cross-cultural adaptation to Brazilian Portuguese of the MCSRS questionnaire and to validate the new version of the tool. The results of this study demonstrate that the judges' opinions were in general agreement on the evaluation of each item within a given criterium, thus confirming the content validity of the instrument. The analysis of the content and face validity of this questionnaire refers to the way in which the translated and culturally adapted instrument can be applied to Brazilian women and understood by them so that its clinical use can be justified. Validity expresses the suitability of the questionnaire, thereby determining whether this measurement tool fulfills the specific purpose for which it was designed. In this report we are presenting the final version to be used for further research.

Patient satisfaction is often used by hospital administrators and health care providers to assess the quality of the medical care provided. The results can guide the planning and development of health services, especially in the context of childbirth care.^17^ Maternal satisfaction has also been associated with the behavior of women in postpartum, promoting maternal and child bonding.^18^ ^19^ To encourage measures that promote women's satisfaction in childbirth is to improve perinatal care, which is one of our objectives, widely shared with others in the field. As a result, childbirth satisfaction surveys have become increasingly common in assessing women's experiences.^20^ ^21^ ^22^ ^23^

It is relevant to address the different validations of this instrument in other cultures, which demonstrates the universality of the method. A Spanish translation and cultural adaptation of the MCSRS questionnaire was performed by Mas-Pons et al,^3^ with the involvement of six experts (obstetricians and midwives). They modified the original version by adding some examples in 10 statements, aiming at better comprehensibility. They also included two items related to the help and support received from the nursing and medical staffs with respect to non-pharmacological methods for pain relief in labor and delivery. In this study, after examination of the forward-translated questionnaire by the panel of 10 experts, an explanation was included in the Brazilian Portuguese version, in parentheses, clarifying the term 'delivery', as 'the moment in which the expulsion of the baby occurred'. Adding other items to the questionnaire was deemed unnecessary. Mas-Pons et al^3^ also applied an adapted version to 10 postpartum women to determine both the amount of time required to answer the questionnaire and the comprehensibility and pertinence of the items in their sociocultural context. In our study, postpartum women also confirmed the face validity of the tool.

Another study^5^ validated the Spanish version of MCSRS. They used the forward translation-back translation method, and the definitive version was achieved after contrasting the opinions of four women. We adopted a similar method to obtain our final version. However, before face validation, we asked 10 experts their opinions on how to better adapt the statements culturally. Subsequently, the opinions of 10 women were used to validate the comprehensibility of the questionnaire's items.

Cultural differences are important for the validation of the MCSRS scale. In validating the Iranian version of MCSRS,^6^ two translators fluent in English and Farsi back-translated the questionnaire, and then an external expert in social sciences compared and reviewed the scale and resolved any discrepancies. Additionally, the questionnaire was presented to 10 mothers for further clarification and wording adjustments. The only structural change was the removal of two items related to the male partner, since fathers are not allowed to be present in labor wards in Iran.

Based on previous studies, the involvement of women in decision-making and control during delivery are factors that influence maternal satisfaction.^24^ ^25^ Such involvement allows mothers to feel empowered and actively participate in childbirth, making the process more pleasant for them. The questionnaire we adapted sought to address this dimension by using Brazilian idioms and transculturally adapted expressions appropriately for Brazilian women. In addition, mothers are in control of their environment, they satisfy their emotional, psychological, and physical needs, and they feel their expectations are being met during labor and delivery.

This study is limited by the fact that it addressed only face and content validity and that its results cannot be extrapolated. There is ongoing research to evaluate the psychometric properties of the Brazilian Portuguese version of the MCSRS questionnaire. Also, since no other valid Brazilian Portuguese questionnaire about childbirth satisfaction was found, the MCSRS could not be compared with similar scales. Another limitation is the lack of items addressing support for the mother in labor, that is not common in Brazil. We would like to have them included in our obstetric practice.^26^

The experts agreed that the items of the final version of the questionnaire meet the evaluation criteria, and the negative correlation is considerably low. The items are adequate and relevant; however, clarity drew slightly less consistent opinions.

## Conclusion

In the present study, the final version of the Brazilian Portuguese MCSRS had its face and content validity confirmed. This instrument seems to be able to accomplish its purpose, namely to measure maternal satisfaction during childbirth. Nonetheless, to use it as an assessment tool, psychometric properties should be further investigated using appropriate methods.
